# The Use of Biologics in Patients with Inflammatory Bowel Disease and Primary Sclerosing Cholangitis

**DOI:** 10.1007/s11901-019-00456-2

**Published:** 2019-03-07

**Authors:** Kate D. Lynch, Satish Keshav, Roger W. Chapman

**Affiliations:** Translational Gastroenterology Unit, Nuffield Department of Medicine, Level 5, John Radcliffe Hospital, University of Oxford, Headley Way, Headington, Oxford, OX3 9DU UK

**Keywords:** Primary sclerosing cholangitis, Inflammatory bowel disease, Ulcerative colitis, Crohn’s disease, Integrin alpha4beta7, TNF-α

## Abstract

**Purpose of Review:**

Biologics are well established in the treatment of many immuno-inflammatory diseases including inflammatory bowel disease (IBD). However, although primary sclerosing cholangitis (PSC) is closely associated with IBD, the role of biologics in PSC remains uncertain. Many new biologics are becoming available to treat IBD, and this review aims to use the experience of biologics in PSC so far to guide more effective evaluation of emerging therapies in the future.

**Recent Findings:**

Antibodies to TNF-α were the first biologics used in IBD, and retrospective analysis suggests that they may have some benefit in PSC, even though an early randomised controlled trial (RCT) showed no effect. Mechanistic studies suggest that TNF-α may have a pathogenic role in PSC. An antibody to integrin α4β7 is effective in IBD, and there are emerging data on its effects in PSC, although no RCT data are available. Mechanistic studies suggest that interrupting the migration of lymphocytes is relevant in PSC. Two biologics, targeting vascular adhesion protein-1 (VAP-1), and lysyl oxidase-like 2 (LOXL2) have been tested in RCTs. The trial of anti-VAP1 is ongoing, whilst the anti-LOXL2 trial was negative.

**Summary:**

Anti-TNF antibodies may benefit PSC when used to treat concomitant IBD, and this may be a direct effect on the liver in a subgroup of patients, or may be an indirect effect of treating IBD. Similarly, anti-integrin therapy may benefit a subset of patients with IBD and PSC. RCTs could decide the role of emerging biologics in PSC, although future trials should be guided by biomarkers that could predict response to the pathway being targeted.

## Introduction

Primary sclerosing cholangitis (PSC) is a progressive inflammatory condition of the liver leading to stricturing and dilatation of the biliary tree. It is closely associated with inflammatory bowel disease (IBD), with approximately 70% of patients with PSC having concomitant IBD [1]. It carries a poor prognosis with mortality rates quoted as high as 28% at 6 years, and currently, there are no effective therapies which can slow its progression [2]. Liver transplantation remains the mainstay of treatment, which is unsatisfactory for obvious reasons [3].

Biologics, which are broadly defined as treatments derived directly or indirectly from living organisms [4], and typically include high molecular weight antibodies and other recombinant proteins, offer the prospect of treating disease by precisely binding to or inactivating a single target in the patient. They differ from conventional medications, which are usually low molecular weight chemicals such as aspirin or mesalazine, in having few side effects caused by interactions with multiple physiological pathways and processes. The increasing use of biologic therapies for chronic immune-inflammatory diseases such as rheumatoid arthritis and IBD has in many cases improved outcomes and reduced the need for surgery [5–9]. However, this therapeutic revolution has so far had little impact on PSC. Nonetheless, because PSC and IBD co-exist in many patients, there is considerable experience of using biologics in patients with both conditions [3, 10].

**Fig. 1:**
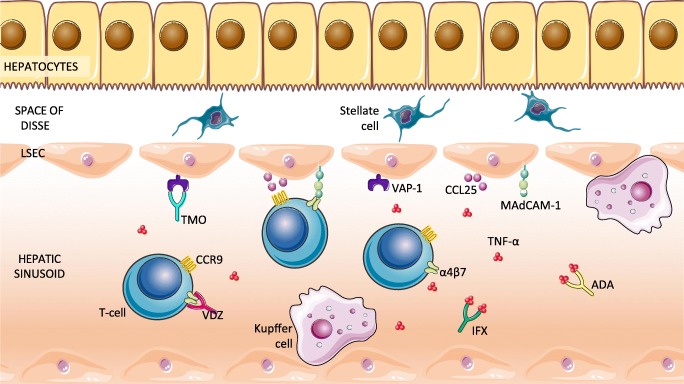
Potential therapeutic drug targets in PSC. Schematic diagram of various potential therapeutic targets of biologic therapy in PSC. The image depicts a hepatic sinusoid, where gut-tropic T cells are slowed down and adhere to the sinusoidal endothelium through the interaction between CCR9 and its cognate ligand, CCL25, as well as binding of a4b7 integrin to MAdCAM-1. VAP-1 also promotes lymphocyte recruitment through multiple mechanisms, including induction of MAdCAM-1 expression. There is an excess of TNF-α seen in PSC. Four monoclonal antibodies are depicted as targeting antigens and interrupting these pathways—infliximab (TNF-α), adalimumab (TNF-α), vedolizumab (α4β7) and timolimumab (VAP-1). ADA, adalimumab; CCL25, chemokine (C-C motif) ligand 25; CCR9, C-chemokine receptor 9; IFX, infliximab; LSEC, liver sinusoidal endothelial cells; MAdCAM-1, mucosal addressin cellular adhesion molecule-1; PSC, primary sclerosing cholangitis; TNF, tumour necrosis factor; TMO, timolimumab; VAP-1, vascular adhesion protein-1; VDZ, vedolizumab. (The authors would like to acknowledge the use of some images from Servier Medical Art in the creation of Fig. 1. https://smart.servier.com/)

The two main biologics that have been used in PSC (for the IBD indication) are anti-tumour necrosis factor-α (TNF-α) therapies and vedolizumab (VDZ), a monoclonal antibody against the integrin, α4β7. Following on from preclinical research suggesting potential benefit specifically for PSC, two other biologics have been tested expressly in PSC: timolimumab, which binds to and blocks vascular adhesion protein-1 (VAP-1); and simtuzumab, which targets the fibrogenic enzyme lysyl oxidase-like 2 (LOXL2). Figure 1 displays these biologic drugs and their targets of action.

The aim of this review is to evaluate the potential role for biologics in PSC by examining preclinical studies which have evaluated the role of TNF-α, α4β7 and related molecules, VAP-1, and LOXL2; and by appraising the clinical literature on biologics that target these molecules in PSC. For the purposes of this review, the use of biologics has only been assessed in patients with PSC prior to liver transplantation.

### Translational Evidence for a Role of TNF-α in PSC

The role of TNF-α is established in the pathophysiology of IBD, and has been exploited by the use of anti-TNF therapy as a beneficial treatment for Crohn’s disease and UC [11]. There is evidence that TNF-α may also play a role in the pathophysiology of PSC. As early as 1992, Spengler et al. created T cell lines from liver biopsies of patients with PSC as well as other liver diseases [12]. After phytohaemagglutinin (PHA) stimulation of these liver T cells, the expression of various cytokines was evaluated within the supernatant by enzyme-linked immunosorbent assay (ELISA), and high expression of TNF-α was demonstrated in PSC as compared with primary biliary cholangitis (PBC). Subsequently, a Swedish study confirmed the same high expression of TNF-α in the supernatant of stimulated lymphocytes from PSC livers as compared with those from healthy controls, PBC and AIH, as well as demonstrating TNF-α intracellularly within the liver T cells by flow cytometry [13].

The potential role of intrahepatic TNF-α in PSC has been investigated in a study by Liaskou et al. [14]. They showed that TNF-α mRNA expression is higher in the PSC liver compared with normal liver at a transcriptional level, and demonstrated that incubation of CD4+ T cells with recombinant TNF-α leads to loss of the expression of the co-stimulatory molecule, CD28 on T cells. CD4+CD28− T cells are chronically active immunopathogenic cells which have been implicated in a range of autoimmune inflammatory conditions [15, 16], and have now been shown to accumulate in the PSC liver, as compared with other diseased livers (primary biliary cholangitis and non-alcoholic steatohepatitis) and especially compared with healthy liver [14]. CD4+CD28− T cells release large amounts of TNF-α on stimulation (as well as IFNγ). This may lead to an autocrine-like induction of further CD28− T cells, as well as TH-1-mediated immune injury to the bile ducts, the site where they have been shown to accumulate [14].

### Published Data on Anti-TNF Therapy in PSC

To date, there are 7 reports of anti-TNF use in PSC, encompassing 107 patients, most of which are retrospective observational case reports or series (see Table 1).

**Table 1:** Characteristics of studies of biologics against TNF-α in PSC/IBD

Study	Year	Location	Design	Treatment	*n*	Liver biochemistry improvement	Comments
Bharuca et al. [17]	2000	Minnesota, USA	Open label	Pentoxifylline*	20	No	Pentoxifylline did not significantly alter symptoms of fatigue or pruritus, serum liver tests, serum TNF-α or TNF-α receptor levels.
Epstein et al. [18]	2005	Massachusetts, USA	Open label	Etanercept	10	No	No patients met primary endpoint (ALP drop > 33%), though 2/10 had small ALP drop. 2/8 patients had resolution of pruritus which returned on discontinuation of drug.
Hommes et al. [19]	2008	Leiden and Amsterdam, Netherlands	Double-blind RCT	Infliximab	10	No	1/6 IFX, 0/4 placebo met primary endpoint (ALP drop > 50% week 18). No difference in change in mean ALP over time in either group. No improvement on liver biopsy histology.
Duca et al. [20]	2013	Vitoria-Gasteiz, Spain	Case report	Infliximab	1	Yes	PSC/IBD patient had ALP drop from 281 IU/L to normal (59 IU/L) This was in conjunction with UDCA and corticosteroids, as well as with IBD resolution.
Franceschet et al. [21]	2016	Padova, Italy	Case series	Adalimumab Infliximab	3 1	Yes No	ALP level assessed at 6- and 12-month post baseline. 2/3 on ADA had ALP drop at month 6 (*n* = 1) month 12 (*n* = 2). Patient on IFX had ALP rise at both time points.
Del Ross et al. [22]	2016	Padova, Italy	Case report	Adalimumab	1	Yes	Psoriatic arthritis with PBC/PSC overlap (no IBD). ALP normalised.
Tse et al. [23•]	2018	Minnesota, USA	Case series	Adalimumab Infliximab	19^a^ 42^a^	Yes No	Evaluated change in mean ALP at 6–8 months and 12–14 months. ADA–ALP drop at both time points (trend only at 12–14 months). IFX–no change in ALP at two time points. No changes in transaminases. Mild rise in direct but not total bilirubin in IFX group. No change in biliary structuring/dilatation on imaging.

Note—only trials involving patients who had not undergone liver transplantation were included

ADA, adalimumab; AISC, autoimmune sclerosing cholangitis; ALP, alkaline phosphatase; CCA, cholangiocarcinoma, CD, Crohn’s disease; CRC, colorectal cancer; IBD, inflammatory bowel disease; IFX, infliximab; LT, liver transplantation; IU/L, international units per litre; MRCP, magnetic resonance cholangiography; PSC, primary sclerosing cholangitis; PC, placebo controlled; *n*, number; RCT, randomised controlled trial; TNF, tumour necrosis factor-α; UC, ulcerative colitis; UDCA, ursodeoxycholic acid

*Note—one drug in table, pentoxifylline, though has mechanistic action against TNF-α, is not itself a biologic

^a^Note—ALP changes were evaluated on a subgroup of these numbers according to availability of biochemistry

Only one double-blind, randomised placebo-controlled trial has been performed by Hommes et al., which evaluated the safety and efficacy of the monoclonal antibody against TNF-α, infliximab (IFX), in patients with PSC and IBD [19]. Patients were randomised 2:1 to receive IFX (5 mg/kg) or placebo according to the usual induction and maintenance schedule for IBD, for a total of 24 weeks, with follow-up to 52 weeks. The primary endpoint was a decrease of serum ALP by 50% or more from baseline to week 18. They also evaluated appearances on liver biopsy at week 0 and 26. The trial was stopped prematurely after an interim analysis of 10 patients (6 having received IFX) due to futility. One patient on IFX met the primary endpoint of ALP drop by > 50% compared with none on placebo, and there was no meaningful change in mean ALP from baseline to week 18 and 52. For the 7 patients for whom paired liver biopsies were available, no change in appearance was seen on histology. Furthermore, 3 patients had to be prematurely withdrawn: one (placebo) due to liver transplantation, one (IFX) due to a dominant stenosis requiring stenting, and another (IFX) due to colorectal cancer. Overall, there was no signal that IFX was beneficial in PSC, and there was a possibility it may be harmful.

The largest report in the literature of anti-TNF therapy in PSC was reported recently in a North American study from the Mayo Clinic [•]. This retrospective observational cohort study evaluated the effect of IFX (*n* = 42) and another monoclonal antibody against anti-TNF, adalimumab (ADA, *n* = 19), in patients with PSC/IBD, evaluating the change in liver biochemistry from baseline to two different time points: 6–8 months and 12–14 months. Whilst there was no difference in mean ALP over time for patients on IFX, there was a statistically significant drop in mean ALP for those on ADA from 249 U/L (SD 158) at baseline to 179 U/L (SD 126) at 6–8 months (*p* = 0.003), and a numerical further drop at 12–14 months to 171 U/L (SD 140) which did not reach statistical significance (*p* = 0.052). There was no change seen in biliary disease on imaging, nor liver stiffness measured via magnetic resonance elastography, as evaluated in a smaller subset. This potential benefit in ADA was also seen in 2/3 patients on ADA in an Italian case series which showed that ALP dropped after 12 months of ADA in 2 of 3 patients (by 12% and 46%) [21]. A case report of a patient with PSC/PBC overlap receiving ADA for psoriasis also showed an improvement in ALP [22].

This small amount of uncontrolled data suggesting a role for ADA but not IFX is intriguing, and if true, the reasons for why one should work and not the other are unclear. One possible explanation is whether the pharmacokinetics of ADA allow it to reach its target of the liver better than IFX due to its larger volume of distribution (up to 6 L as compared with 3 L for IFX) [23].

There are two open-label studies of anti-TNF agents that have been evaluated in PSC [17, 18]. One is pentoxifylline, an oral xanthine derivative, which is therefore not a biologic, tested in 20 patients [17]; the other is etanercept, a subcutaneously delivered fusion protein that is licenced for use in rheumatoid arthritis (though notably has no benefit in Crohn’s disease), that was tested in 10 patients [18, 25]. Both of these studies showed no significant benefit on liver biochemistry, though interestingly, etanercept resolved pruritus in 2/8 patients, which returned on its discontinuation.

Taking these data together, whilst there is evidence for a possible role of TNF-α in the pathophysiology of PSC, the reported literature on anti-TNF therapy in PSC is disappointing. The efficacy endpoints have not been reported in a consistent manner, and the heterogeneity of the phenotype in PSC makes it difficult to draw clear conclusions from studies and reports involving such small numbers. One possibility that there has not been robust evidence for anti-TNF therapy in PSC is the concept of an “antigen sink” [26]. The clearance of anti-TNF monoclonal antibodies, particularly infliximab, is influenced by the amount of TNF-α present both in the blood and in tissue (soluble and membrane-bound). Higher levels of TNF-α occur with increased inflammatory burden [27]. Therefore, in active IBD, high levels of antigen (TNF-α) lead to formation of immune-complexes with infliximab/ADA, leading to phagocytosis and increased clearance. It is possible that in active IBD, the anti-TNF is not able to reach high enough concentrations within the liver to have an effect, and possibly higher doses of therapeutic antibody are required.

There may be a subtype of PSC which is more likely to respond to anti-TNF therapy, and identifying these subgroups illustrated in a few of these studies should be a goal of any future studies. A large collaborative effort is currently underway by the International PSC Study Group (IPSCSG) to describe the experience in a large international cohort of patients with PSC/IBD on anti-TNF therapy. Meanwhile, there is a signal that ADA may be beneficial in PSC in a way that IFX is not, and ADA should be investigated with a prospective well-stratified clinical trial, particularly with the forthcoming availability of biosimilars and therefore reducing cost of the drug.

### Translational Evidence for Targeting Gut-Homing Lymphocytes in PSC

Given the strong relationship between PSC and IBD, it is not surprising that there is evidence for a link between immune cells originating from the gut being involved in the pathophysiology of PSC.

The majority of T cells within the intestine have a particular phenotype which causes them to home from the peripheral circulation to the gut. In particular, they express the chemokine receptor, CC chemokine receptor 9 (CCR9), as well as the integrin α4β7, in response to encountering bacterial antigens presented in mesenteric lymph nodes [28–30]. Circulating CCR9+ α4β7+ T cells are recruited to the gut through binding of its ligand, chemokine (C-C motif) ligand 25 (CCL25), which is expressed on the intestinal vascular endothelium. This binding causes the T cell to slow down and roll along the endothelium and it also triggers the activation and upregulation of further α4β7 integrin to the cell surface. α4β7 then binds to mucosal addressin cellular adhesion molecule-1 (MAdCAM-1), which is also expressed on the intestinal vascular endothelium. This binding arrests the T cell in its motion, and it subsequently enters the lamina propria of the intestine.

The first evidence for this particular type of intestine-specific cell being important in PSC came from a human translational study from Birmingham, UK [31]. Grant et al. demonstrated that MAdCAM-1 was also expressed in the liver, and was particularly localised to the portal veins, as identified by immunohistochemistry. They showed that there was a greater hepatic expression of MAdCAM-1 in PSC (and autoimmune hepatitis) relative to other chronic liver diseases. Furthermore, they showed through tissue-based assays that α4β7+ T cells can bind to hepatic MAdCAM-1 through the action of CCL25, which can be blocked with antibodies against α4β7 and MAdCAM-1.

The group then demonstrated that there was a significant population of gut-phenotypic T cells within the human liver in PSC as compared with other liver diseases or healthy liver donors [32]. They demonstrated via flow cytometry that 20% of PSC liver-infiltrating T cells were positive for CCR9 (most of which were also positive for α4β7) compared with less than 2% of control liver-infiltrating T cells. Furthermore, there was strong expression of its ligand, CCL25 in the PSC liver compared with controls, as demonstrated by Western blotting and quantitative PCR. This higher expression of these gut-derived T cells present in the PSC liver was validated in a study some 10 years later by Liaskou et al. [14].

The question of whether these gut-phenotypic T cells within the PSC liver are derived from the gut or actually primed with CCR9 and α4β7 within the liver (and hence, did not come from the gut at all) was answered in a further study by Eksteen et al. [33]. They co-cultured naïve CD8+ T cells with hepatic dendritic cells (DCs), portal lymph node DCs, stellate cells, or intestinal DCs. Only those cultured with intestinal DCs induced high levels of expression of CCR9 and α4β7, and there was minimal to no effect with the hepatic/portal-derived cells. These studies included cells derived from human PSC livers. Hence, it was concluded that the CCR9+ α4β7+ T cells seen within the PSC liver have their origins in the intestine.

Subsequently, a collaborative study was performed between the UK (Birmingham) and the Norwegian PSC Research Center [34]. They carried out high-throughput sequencing of T cell receptor β repertoires from matched colon, liver and blood samples from patients with PSC/IBD and normal gut and liver samples from colon cancer patients. There was a significantly higher overlap between the gut and liver of T cell clones from PSC/IBD patients (mean 8.7%) compared with controls (3.6%). This difference must be borne against the fact that the PSC/IBD tissue was fresh-frozen tissue compared with the control tissue which was formalin-fixed, paraffin-embedded (FFPE). Whether the formalin and/or paraffin had any bearing on breakdown of T cells or their receptors which could confound this finding is unclear. Nevertheless, the implication is that there are more shared characteristics between gut and liver T cells in PSC/IBD compared with so-called healthy controls, lending further support for a role of gut-derived T cells in the pathogenesis of PSC.

Lastly, a role for a related molecule, vascular adhesion protein-1 (VAP-1), has been put forward [35••]. VAP-1 is constitutively expressed on sinusoidal endothelial cells in the liver, and, through its enzymatic activity, can assist with lymphocyte recruitment via induction of MAdCAM-1 expression by endothelial cells [36–38]. Hepatic VAP-1 expression has been shown to be greater in diseased liver as compared with healthy liver, and to the greatest extent in PSC [35••]. Furthermore, it has been shown that VAP-1 enzymatic activity is elevated in PSC versus normal liver, and that this activity promotes adhesion of α4β7+ lymphocytes to the hepatic endothelium [35••]. VAP-1 exists also in a soluble form (sVAP-1), and not only have higher sVAP-1 levels been reported in PSC versus controls, but within PSC patients, a higher sVAP-1 level is associated with a worse clinical outcome (that is, reduced transplant-free survival) [35••].

Multiple biologic drugs exist which are capable of therapeutically targeting this pathway and may be exploited for this purpose in PSC. A phase II open-label trial is currently underway in the UK evaluating the efficacy of a monoclonal antibody, timolimumab, against VAP-1 in PSC [39].

There is also a licenced monoclonal antibody against α4β7, VDZ, which binds to α4β7 and prevents binding to its ligand MAdCAM-1, thereby inhibiting lymphocyte recruitment. VDZ has efficacy in UC and Crohn’s disease, and observational data in PSC is reviewed below. There are also further therapeutics in development which target MAdCAM-1 and CCR9 which could also play a role in PSC.

### Published Data on Anti-integrin Therapy in PSC

There are 5 published case reports/series [23•, 40–42, 43•], and 4 case series in abstract form only [44–47], evaluating the effect of VDZ in PSC/IBD, comprising a total of 211 patients (see Table 2). These studies vary once again with regard to what endpoints they report on. Some are purely descriptive, many evaluate the liver biochemistry at various time points, particularly the ALP, and some report the effect of VDZ on the IBD itself.

**Table 2:** Characteristics of studies of vedolizumab in PSC/IBD

Study	Year	Location	Design	Treatment	*n*	Liver biochemistry improvement	Comments
Lim et al. [40]	2016	London, UK	Case series	Vedolizumab	10^b^	Unknown	Included patients with AISC and PSC. 4/10 (40%) had clinical response of IBD. Liver biochemistry was not formally evaluated.
Westerveld et al. [41]	2017	Florida, USA	Case report	Vedolizumab	1	Yes	ALP improved from 225 to 127 at 13 months on VDZ. Transaminases also improved. Inflammation and biliary stricturing improved on MRCP.
Coletta et al. [42]	2017	Milan, Italy	Case report	Vedolizumab	1	No	Patient had PSC/UC with ileal pouch anal anastomosis. Clinical and endoscopic remission of pouchitis. Correlated with increase in circulating α4β7 + memory CD4+ T cells.
Christensen [43•, 48]	2018	Chicago, Wisconsin, and Michigan, USA and Melbourne, Australia	Case series	Vedolizumab	34^a,b^	Mixed response	Overall (ALP available for *n* = 26), no significant change in ALP from baseline (268 IU/L) to week 30 (249 IU/L, *p* = 0.99). In patients with ALP > ULN at baseline, ALP reduction at week 14 but not week 30 – may be confounded by concomitant UDCA. Clinical remission of IBD at week 30 in 55% for CD, 29% for UC. 1/28 (3.6%) pre-LT patients required LT whilst on VDZ. 2/34 (5.8%) developed cholangitis within 6 months VDZ.
Tse et al. [23•]	2018	Minnesota, USA	Case series	Vedolizumab	27^a^	No	Non-significant rise of baseline ALP from 260 IU/L to 310 IU/L at month 6–8 (*p* = 0.11) and 319 IU/L at month 12–14 (*p* = 0.24). No change in transaminases. No change in biliary structuring/dilatation on imaging.
Williamson, et al. [44]	2017	Oxford, UK	Abstract: Case series	Vedolizumab	11	No	Trend for ALP rise over mean duration 206 days but not to statistical significance. Improved UC endoscopic score (UCEIS) from 4.4 to 2.7, *p* = 0.04. Improvement in IBD corresponded with AL drop and vice versa. Colonic expression of β7 on T cells reduced in colon with VDZ.
Caron, et al. [45]	2018	22 centres in Belgium and France	Abstract: Case series	Vedolizumab	54	Yes in small proportion	Primary outcome was decrease of serum ALP by ≥ 50% from baseline to week 30. 4/54 (7.4%) reached primary endpoint at week 30. 3/37 (9.1%) achieved ≥ 50% ALP reduction at week 54. Overall, mean ALP level increased by 0.4 × ULN at week 54. No change in transaminases observed. 5/54 (9.2%) developed cancer (3 × CRC, 2 × CCA).
Doherty, et al. [46]	2018	Dublin, Ireland	Abstract: Case series	Vedolizumab	13^b^	No	Statistically significant rise at several time points in median ALP from week 0 (126 IU/L) through to week 36 (190 IU/L, *p* = 0.028). Showed no significant ALP rise in IBD alone cohort (i.e. no PSC) on VDZ (*n* = 31). Median faecal calprotectin improved from baseline to month 6.
Williamson et al. [47]	2018	11 centres in North America and Europe	Abstract: Case series	Vedolizumab	60	Yes in half Overall rise	Overall, non-significant rise in mean ALP from 2.38 × ULN at baseline to 2.59 × ULN day 42 (*p* = 0.32) Statistically significant rise to 2.76 × ULN at last follow-up (median 363 days, *p* = 0.06). 50.9% had an ALP drop compared with baseline at last follow-up (mean drop − 22.6%). Rise in mean ALT: baseline 61.6 IU/L vs. 79.8 IU/L, *p* = 0.0078. IBD improved endoscopically in 25/44 (56.8%).

Note—only trials involving patients who had not undergone liver transplantation were included

ADA, adalimumab; AISC, autoimmune sclerosing cholangitis; ALP, alkaline phosphatase; CCA, cholangiocarcinoma, CD, Crohn’s disease; CRC, colorectal cancer; IBD, inflammatory bowel disease; IFX, infliximab; LT, liver transplantation; IU/L, international units per litre; MRCP, magnetic resonance cholangiography; PSC, primary sclerosing cholangitis; PC, placebo controlled; *n*, number; RCT, randomised controlled trial; UC, ulcerative colitis; UCEIS, ulcerative colitis endoscopic index of severity; UDCA, ursodeoxycholic acid; VDZ, vedolizumab

^a^Note—ALP changes were evaluated on a subgroup of these numbers according to availability of biochemistry

^b^This is the total number of patients analysed, a portion of which included some patients receiving VDZ post liver transplantation

Whilst descriptive, one of the case reports is interesting. An American case report from 2017 of a male with PSC/UC was given VDZ for 13 months after his ALP remained persistently elevated following a trial of UDCA [41]. Subsequently, his ALP normalised from 225 to 127 IU/L and follow-up MRCP showed improvement in biliary stricturing.

The two largest published case series come from the Mayo Clinic in USA (*n* = 27) [23•] and a North American/Australian collaboration (*n* = 34) [43•]. It is important to note here that whilst 27 and 34 patients were included in these analyses respectively, liver biochemistry parameters and other endpoints were only available among a subset of these patients. Both studies evaluated ALP and other liver biochemistry parameters at baseline and two later time points (month 6–8 and month 12–14 for one study, and week 14 and week 30 for the other). When evaluating the mean/median ALP in the overall cohort, there was no significant change in ALP, transaminases or bilirubin from baseline to any time point in either study.

In one study, they split patients up into two cohorts—those with elevated baseline ALP (*n* = 18) and those with normal baseline ALP (*n* = 8) [43•]. In the first subgroup (raised ALP at baseline), there was a statistically significant drop in median ALP from baseline (475 IU/L) to week 14 (322.5 IU/L, *p* = 0.025) and a further numerical non-significant drop at week 30 to 283 IU/L (*p* = 0.267). Another way of looking at this, is that 11/18 (69%), after initially starting with elevated baseline ALP, had an improvement in ALP on VDZ (though none actually completely normalised their ALP). Conversely, among those patients with normal baseline ALP, half (4/8) had a rise in their ALP, with an overall small but statistically significant rise in median ALP.

The reason for patients with a raised ALP at baseline potentially benefiting from VDZ is unclear. It may be that this subset of patients has a higher inflammatory burden, and therefore augmenting hepatic lymphocyte recruitment may have a beneficial effect. These data are the first to suggest a possible subset of patients with PSC who may benefit from VDZ, an idea which ought to be further explored in larger prospective studies.

Whilst the other large study did not look at predictors of ALP drop, they did examine whether VDZ has any effect on radiological evaluation of biliary stricturing as well as on liver stiffness as measured by MR elastography (the latter in a very small subset), both measured at 1 year. Unfortunately, neither showed any improvement, though this is hardly surprising, as VDZ is expected to have an anti-inflammatory reaction but it would be very unlikely for it to have an antifibrotic effect. Its method of action would be suspected to slow progression to fibrosis and stricturing rather than reverse the process altogether.

Whilst currently not in peer-reviewed manuscript form, the published abstracts offer further interesting possibilities of whether VDZ may be effective in PSC and whether there are certain subgroups which stand to benefit more [44–46].

Two of the abstracts involved rather larger cohorts—the French GETAID cohort [45] analysed 54 patients whilst the International PSC Study Group (IPSCSG) examined 60 patients [47]. All four abstracts showed an overall rise in mean/median ALP from baseline to a later time point on VDZ, and in 2/4 abstracts, this rise was statistically significant. The proportion the ALP rose by varied from 16 to 75% from baseline, but in the largest two cohorts, the rise was 16% in one cohort (at median 363 days) and 18% in the other (at week 30). Both of these rises could arguably be in keeping with the natural history of the disease.

The primary endpoint of the French GETAID group was a very strict one: ALP reduction by ≥ 50% from baseline. 7.4% achieved this by week 30, and 9.1% by week 54. Some might argue that a smaller drop is clinically significant, or potentially a decrease below a certain threshold, such as < 1.5 × ULN, which has been shown to be associated with a better clinical prognosis [49–52].

In the case of the IPSCSG study, the median ALP rose in a proportion of patients, and fell in another subset. They found that 50.9% of patients had had a decrease in ALP by last follow-up with a median drop of 22.6%. It is possible that these divergent responses reflect subgroups of patients with opposite responses to treatment. Therefore, the overall response, which was a minor rise in ALP, should be interpreted with caution.

**Fig. 2:**
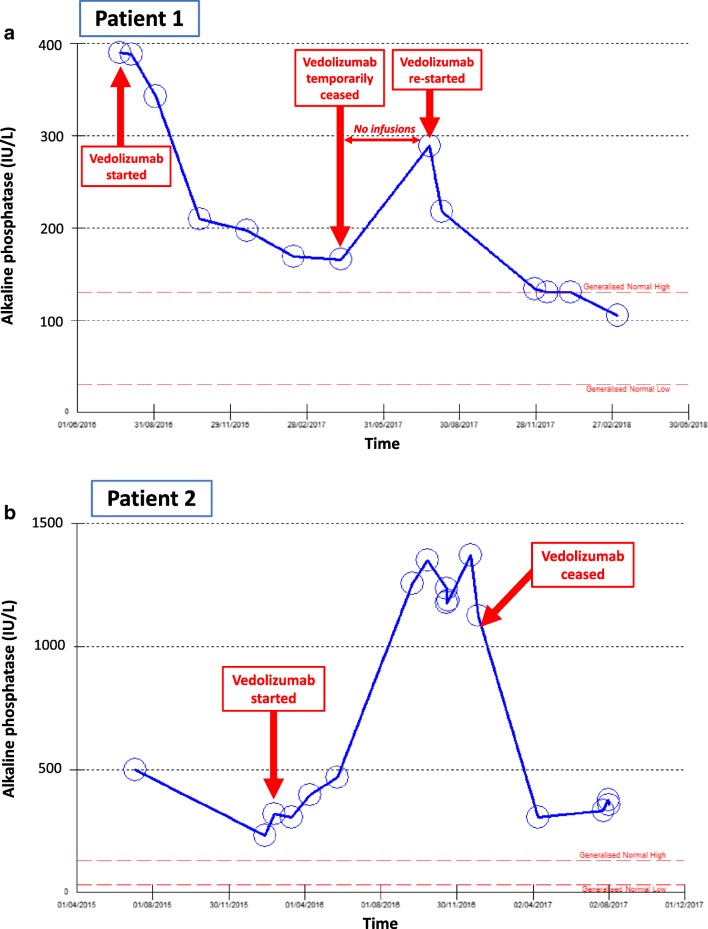
Differing ALP response to VDZ in two patients with PSC/UC. These graphs show the serum ALP level over time in two individual patients with PSC who were commenced on VDZ for their underlying UC. Both patients received VDZ according to the usual schedule as licenced with induction and maintenance. The dotted red lines show the ULN and LLN for ALP at our institution. Each open blue circle represents a measurement of serum ALP. **a** This patient did not attend for two of his infusions part way through his therapy, and so VDZ was inadvertently temporarily ceased. Upon recommencement, he received induction dosing again before maintenance dosing. He went into clinical and endoscopic remission of his underlying UC. **b** This patient remained on VDZ for 5 months before a decision was taken to cease the medication, as it had had no effect on his UC, both clinically and endoscopically. ALP, alkaline phosphatase; LLN, lower limit of normal; PSC, primary sclerosing cholangitis; UC, ulcerative colitis; ULN, upper limit of normal; VDZ, vedolizumab

These explanations are purely speculative, and it is clear that if a study were to be performed prospectively of VDZ in PSC, stratification would be key to understanding the potential effect of VDZ in this disease. Personally, in our institution, a patient with PSC/IBD has been observed who had an immediate fall in ALP levels on commencement of VDZ, only to rise again on interruption of the VDZ then fall once more on readministration of the drug (see Fig. 2a). Conversely, another of our patients had a significant rise in their ALP (and ALT) coinciding with commencing VDZ, despite a liver biopsy showing no significant drug interaction nor interface hepatitis (see Fig. 2b).

Thus, reverse translational proof of concept studies, looking at how VDZ alters the immune populations and phenotype within the gut and the liver, would also be useful, and potentially may provide biomarkers to identify patients who could benefit from therapy with VDZ. This could involve sampling the colon and liver prior to and during VDZ therapy and analysing certain markers such as expression of α4β7 and related chemokine receptors and integrins as predictors of response.

Finally, it is important to point out that in 5/9 studies/abstracts mentioned, response of the IBD in PSC to VDZ was reported. In all of these cases, there was a signal that a significant proportion of patients responded as compared to those with IBD alone, whether it be analysed clinically, by faecal calprotectin levels or endoscopically. Given that there is no clear signal from these studies that VDZ is harmful to patients with PSC/IBD, if a patient with PSC/IBD has an indication for VDZ with regard to their IBD, it is appropriate to initiate VDZ in this cohort.

### Translational Evidence for a Role of Lysyl Oxidase-like 2 in PSC

Lysyl oxidase-like 2 (LOXL2) is an enzyme which catalyses the cross-linkage of collagen and elastin, thereby stabilising the fibrotic matrix [53]. In Mdr2^−/−^ mouse models, a common animal model used in PSC research which exhibits biliary fibrosis, LOXL2 inhibition by an anti-LOXL2 antibody slowed fibrosis progression and reduced hepatic stellate cell activation [54]. A recent study then showed that serum levels of LOXL2 are significantly higher in PSC (and also PBC and secondary sclerosing cholangitis) compared with healthy controls [55]. Furthermore, immunochemical staining of PSC livers showed localisation of LOXL2 in the fibrous septa and periductal onion-skin type fibrosis. These findings suggest that therapeutically targeting LOXL2 may be beneficial in humans with PSC.

### Published Data on Anti-LOXL2 Therapy in PSC

Simtuzumab is a monoclonal antibody against LOXL2, for which a large phase 2B blinded randomised placebo-controlled trial has been carried out in PSC [56•]. This demonstrated no beneficial effect of two different doses of simtuzumab over placebo on the primary outcome of mean change in hepatic collagen content at week 96, as assessed by morphometry on liver biopsy specimens. Whilst the trial was negative, the well-conducted study generated a wealth of knowledge about the natural history of PSC and useful evaluation of various non-invasive fibrosis markers over time.

It is worth noting that simtuzumab has been trialled in a variety of fibrotic conditions, as well as a couple of malignant conditions, with unfortunately no signal for clinical efficacy [57–62]. These conditions include a large phase 2B study of almost 500 patients with non-alcoholic steatohepatitis with bridging fibrosis or cirrhosis, where 96 weeks of simtuzumab had no effect over placebo upon hepatic collagen or fibrosis stage [62]. Despite the promise of targeting LOXL2 from translational research, it appears simtuzumab will not be beneficial in PSC and other fibrotic liver conditions.

## Conclusion

Biologics have transformed the treatment of IBD, and because physicians caring for patients with IBD frequently also treat concomitant PSC, they have long provided hope and interest in the PSC community. However, apart from recent experience with simtuzumab and timolimumab, there have been few RCTs looking specifically at PSC. Nonetheless, detailed analysis of preclinical and mechanistic studies, and experience of the use of anti-TNFs and vedolizumab in PSC/IBD provides some cause for cautious optimism. It may be that improving inflammation in the intestine can have some benefit on liver chemistry in PSC, at the very least, or that there is some direct benefit of VDZ and ADA, at minimum. These opportunistic, retrospective studies of the effect of biologics used for IBD, in patients with IBD and PSC, however, are inadequate to guide therapy in the future, particularly as more and more biologics targeting potentially important targets such as IL-23, IL-22, and IL-17 become available. Opportunistic studies lack power, stratification, and because they are typically retrospective, are subject to confounding.

There are some clues that ADA and VDZ may have some benefit in a subset of patients, but meaningful conclusions cannot be drawn without the aid of well-stratified prospective clinical trials. The addition of human proof of concept studies as side arms to these trials, incorporating collection and evaluation of tissue samples to identify biomarkers for response, would add significant weight and mechanistic detail to any clinical data collected, and should be considered in clinical trials in the future. This approach might allow PSC treatment to be an exemplar of personalised treatment.
